# Complete mitochondrial genome of chestnut-fronted macaw (*Ara severus*, Psittaciformes)

**DOI:** 10.1080/23802359.2016.1250131

**Published:** 2017-01-17

**Authors:** Adam Dawid Urantówka, Aleksandra Kroczak, Paweł Mackiewicz

**Affiliations:** a Department of Genetics, Wroclaw University of Environmental and Life Sciences, Wroclaw, Poland;; b Department of Genomics, Faculty of Biotechnology, Wrocław University, Wrocław, Poland

**Keywords:** Psittaciformes, *Arini*, mitogenome, *Ara severus*, chestnut-fronted macaw

## Abstract

Six genera from *Arini* tribe form a morphologically diverse group named macaws, which differ from other *Arini* in the presence of bare facial area. Macaws are further distinguished by the bare face pattern, plumage colouration and body size. Six of the eight macaw species from *Ara* genus can be easily segregated into three pairs according to their colouration. An exception is *Ara severus*, which differs from others in their size and morphology. The lack of appropriate molecular markers precludes determination of its phylogenetic position. Therefore, the mitogenome of *Ara severus* presented in this report will be indispensable to refine these phylogenetic relationships.

According to the most recent taxonomy of parrots, all neotropical species are classified into *Arinae* subfamily (Joseph et al. 2012). Among the four tribes recognized within this subfamily, the *Arini* tribe is the most taxon-rich and morphologically diversified (Schweizer et al. 2014). The majority of 19 extant genera recognized within this tribe are classified as macaws (with six genera) or conures (with nine genera) (Remsen et al. 2016). Both conures and macaws are slender birds with broad wings; long, graduated tails and heavy bills (Forshaw 2010). However, the presence of bare facial area distinguishes macaws from conures and other *Arini* members. Macaws are further diversified in the pattern of this bare skin and in body size. The smallest one is red-shouldered macaw (*Diopsittaca*). *Orthopsittaca* and *Primolius* are of medium size, whereas *Anodorhynchus* and *Cyanopsitta* include big forms. Seven *Ara* species are also large-bodied parrots, but one species, *Ara severus* has a medium size. This genus is also much diversified according to plumage. Six of the *Ara* species can be segregated into three pairs according to this feature: *Ara ararauna* and *Ara glaucogularis* have blue and yellow colouration; *Ara macao* and *Ara chloroptera* show predominantly red or scarlet colouration; *Ara militaris* and *Ara ambigua* are characterized by predominantly green colouration. Despite the green colour that prevails also in the plumage of the two other *Ara* species *rubrogenys* and *severus*, they significantly differ in colouration of forehead, crown, ear-coverts, throat, shoulders, lesser, and median wing-coverts, lesser and greater underwing coverts, primaries, secondaries, and tails (Juniper & Parr 1998). These species have also a considerably different pattern of bare facial areas typical of macaw species. In the medium-sized *Ara severus*, the extent of bare facial area resembles the pattern characteristic of typical large *Ara* species. On the other hand, the bare facial region of large *Ara rubrogenys* species is reduced nearly to lores, where only single black feathers are present.

Taking into account the mixed nature of the morphological characteristics of *Ara severus* and *Ara rubrogenys*, it is impossible to determine their phylogenetic position within other *Ara* species based only on morphology. Moreover, there are no appropriate molecular markers that could resolve the phylogenetic relationships between them. Therefore, we sequenced *Ara severus* mitogenome with the length of 16,994 bp (under GeneBank accession number KF946546) to gain appropriate molecular data for future examination of evolutionary diversification of macaws.

Although morphology of the analyzed specimen, being Polish captive bird, was undoubtedly typical of *Ara severus*, we compared its control region sequence with all available control region sequences from other 12 macaws to prove its species belonging. The obtained tree (Figure 1) revealed that the analyzed *A. severus* individual groups significantly with two other representatives of this species. Its control region sequence shows 99.8% and 99.5% identity with them. *Ara severus* group is sister to the clade including *Ara ararauna* and *Ara glaucogularis*, which share the same plumage colouration. However, species with similar body size are separated in the tree implying independent changes in this feature during evolution.

**Figure 1.**
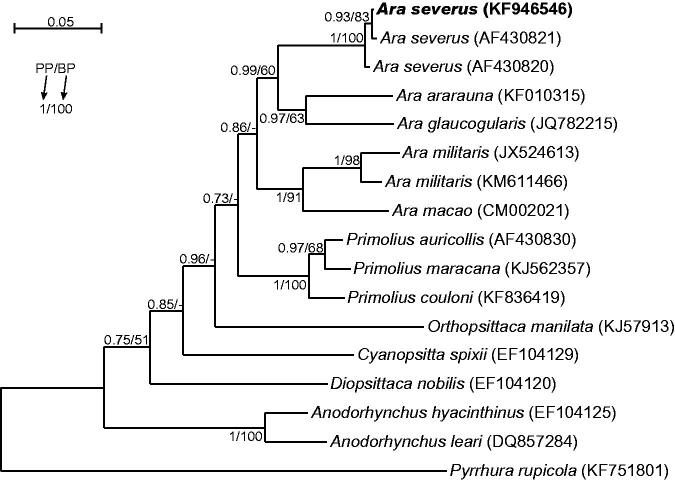
The phylogenetic tree obtained in MrBayes for control region sequences indicating that the studied individual (bolded) belongs to *Ara severus* species. Its blood sample from which DNA was isolated is available in the laboratory at the Department of Genetics in Wroclaw University of Environmental and Life Sciences under the number Arasev16994. Values at nodes, in the order shown, indicate posterior probabilities found in MrBayes (PP) and bootstrap percentages calculated in PAUP* (BP). Bootstrap percentages <50 were indicated by a dash ‘-’. In the MrBayes (Ronquist et al. 2012) analysis, we assumed a mixed substitution model with gamma-distributed rate variation across sites as proposed in jModelTest (Darriba et al. 2012) based on BIC criterion. We applied two independent runs, each using four Markov chains. Trees were sampled every 100 generations for 10,000,000 generations. After obtaining the convergence, trees from the last 6,028,000 generations were collected to compute the posterior consensus. In the case of PAUP* (Swofford 1998), trees were searched in bisection and reconnection (TBR) branch-swapping algorithm and TPM2u + Γ substitution model was applied as found in jModelTest among 1624 candidate models according to BIC criterion. In bootstrap analysis, 1000 replicates were assumed. Five discrete rate categories were assumed for gamma-distributed rate variation across sites.
